# A Hot-Polymer Fiber Fabry–Perot Interferometer Anemometer for Sensing Airflow

**DOI:** 10.3390/s17092015

**Published:** 2017-09-02

**Authors:** Cheng-Ling Lee, Kai-Wen Liu, Shi-Hong Luo, Meng-Shan Wu, Chao-Tsung Ma

**Affiliations:** 1 Department of Electro-Optical Engineering, National United University, Miaoli 360, Taiwan; ccpsandy0127@gmail.com (K.-W.L.); u0323144@gm.nuu.edu.tw (S.-H.L.); ynes2367@gmail.com (M.-S.W.); 2 Department of Electrical Engineering, National United University, Miaoli 360, Taiwan; ctma@nuu.edu.tw

**Keywords:** fiber-optic sensors, fiber-optic components, Fabry–Perot, polymers, optical sensing and sensors, interferometry

## Abstract

This work proposes the first hot-polymer fiber Fabry–Perot interferometer (HPFFPI) anemometer for sensing airflow. The proposed HPFFPI is based on a single-mode fiber (SMF) endface that is attached to a UV-cured polymer to form an ultracompact fiber Fabry–Perot microcavity. The proposed polymer microcavity was heated using a low-cost chip resistor with a controllable dc driving power to achieve a desired polymer’s steady-state temperature (T) that exceeds the T of the surrounding environment. The polymer is highly sensitive to variations of T with high repeatability. When the hot polymer was cooled by the measured flowing air, the wavelength fringes of its optical spectra shifted. The HPFFPI anemometers have been experimentally evaluated for different cavity lengths and heating power values. Experimental results demonstrate that the proposed HPFFPI responses well in terms of airflow measurement. A high sensitivity of 1.139 nm/(m/s) and a good resolution of 0.0088 m/s over the 0~2.54 m/s range of airflow were achieved with a cavity length of 10 μm and a heating power of 0.402 W.

## 1. Introduction

The development of flow measurement techniques is important in industry and scientific technology. Many measurement techniques that use anemometers and flowmeters have been developed, but the technique of hot-wire anemometry (HWA) is still the preferred approach to flow sensing because it has many advantages and, in particular, can be used to measure rapid flows [1]. The measuring mechanism of HWA is based on the heat transfer from sensors to the surrounding environment; the method is simple and reliable. Therefore, fiber-optic anemometers based on HWA have attracted substantial research interest [2,3,4,5,6,7,8,9,10,11,12]. The most common fiber anemometer based on HWA is the well-known heated fiber Bragg grating (FBG) [2,3,4,5,6,7,8,9,10,11]. Other sensing configurations that are based on the HWA mechanism, such as those in the intermodal interferometer [12] and the Fabry–Pérot interferometer [13], have been proposed. The temperature of heated FBGs depends on the velocity of flow of the surroundings, which cools the FBGs, shifting their spectral resonance peaks. To heat the FBG, Lamb et al. firstly proposed a heating technique that relies on a remote infrared CO_2_ laser beam to heat a small region of the FBG [11]. The active-heating FBG method is very convenient, but precise alignment of the FBG and laser is required, and the laser is very bulky. To simply obtain a hot fiber section in an FBG, the attenuation fibers and active fibers are used to fabricate the FBG to achieve a passive HWA configuration in the FBGs [2,3]. However, actively controlled FBGs that are based on direct self-heating by an in-fiber light/laser have been demonstrated to be effective [4,5,6,7,8,9,10,11]. Active FBG-HWAs coated with metallic (Au or Ag) thin films to greatly absorb heating light can increase the efficiency of heating [5,6,7,8,9,10]. The metal-coated FBG-HWAs can be further combined with a no-core fiber [6], bitaper [7], core offset fiber [8], and long-period fiber grating [5] to help coupling the heating light into the fiber cladding so as to enhance the absorption of heat by the metallic thin films. The above sensing schemes are highly practical and efficient, but the expensive laser writing the FBGs, the metallic vacuum coating, and the high-power laser that heats the FBGs make the fabrication of FBG-HWAs time-consuming and complicated. Zhao et al. therefore developed a non-FBG-HWA gas flowmeter that is based on intermodal interference to improve on the low thermal sensitivity and non-uniform heating effect in the aforementioned FBG-HWAs [12]. The non-FBG-HWA configuration provided greater sensitivity than that of the FBG-HWAs, but a high vacuum metallic coating procedure was still necessary [12].

This paper develops the first tiny, simple, flexible, non-FBG anemometer that is based on a hot-polymer fiber Fabry–Perot interferometer (HPFFPI). The proposed HPFFPI anemometer with the NOA61 polymer that is attached to its SMF endface swells or shrinks as the ambient T increases or decreases and red-shifts or blue-shifts the interference spectrum. Since the polymer is highly sensitive to variations in T with high repeatability, the proposed polymer cavity was initially heated using a tiny chip resistor with a fixed driving power that exceeds the T of the surroundings. The heating method is straightforward, low-cost, and different from that of hot FBG-based anemometers that are heated using high-power lasers. The hot polymer can be cooled by the wind, shifting the wavelength fringes of the interference spectra. Experimental results demonstrate that the proposed HPFFPI responds with good sensitivity and high resolution to variations in the airflow speed.

## 2. Configuration and Operating Principles

**Figure 1:**
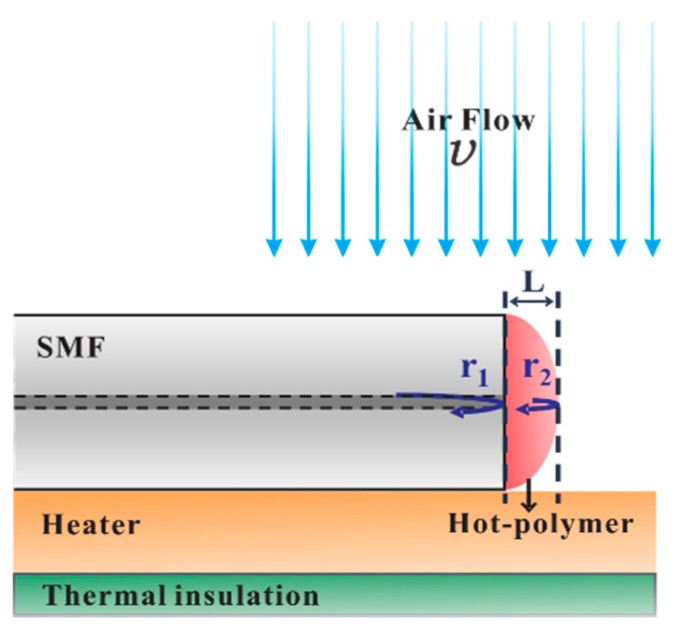
Proposed hot-polymer fiber Fabry–Perot interferometer (HPFFPI) anemometer.

**Figure 2:**
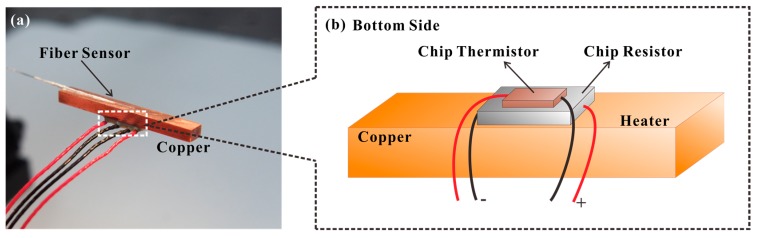
(**a**) Proposed low-cost chip resistor as a heating element; (**b**) bottom of the heating element.

Figure 1 presents the sensing element of the proposed hot-polymer-based anemometer. Exploiting the favorable T-responsive characteristics of the proposed HPFFPI, the sensor is initially packaged and heated to a high T that is greater than the ambient T. The heated polymer is cooled by the wind, shifting its wavelength fringes. The sensor is configured as a simple kind of fiber Fabry–Perot interferometer, and its interferometric mechanism is based on two-beam interference with low finesse [14]. The polymer NOA61 (n_D_ = 1.56) forms a microcavity that generates low-finesse interference by reflecting from the first and second interfaces. Monitored translation stages are used to simply attach the thick film of NOA61 onto the endface of an SMF and form the fabricated HPFFPI probe. One can repeat the steps of coating and UV-curing several times to obtain a desired cavity length. The NOA61 is a common clear, colorless, and liquid photopolymer. It can be cured by exposure to ultraviolet (UV) light, which transforms liquid NOA61 into a full solid with a strongly bonded structure. Thereafter, the aging maximizes the strength of the adhesion of NOA61 to the fiber and stabilizes the performance [15]. Herein, the chip heater, used to heat the polymer, is a homemade device that is fabricated by gluing a chip thermistor with a chip resistor to a small strip of copper (Cu) (25 mm × 3 mm × 2 mm), as shown in Figure 2a. The proposed fiber anemometer was glued using thermal conduction gel on the copper, whose temperature was monitored and controlled using the chip thermistor and chip resistor that was glued to the back of the copper, as depicted in Figure 2b.

A standard dc power supply with a controllable output current was used to adjust the desired steady-state T of the strip of copper with different output power levels. The fabrication of the HPFFPI sensor and the sensing system is very simple, flexible, and low-cost, and it is believed that these devices have great commercial potential.

**Figure 3:**
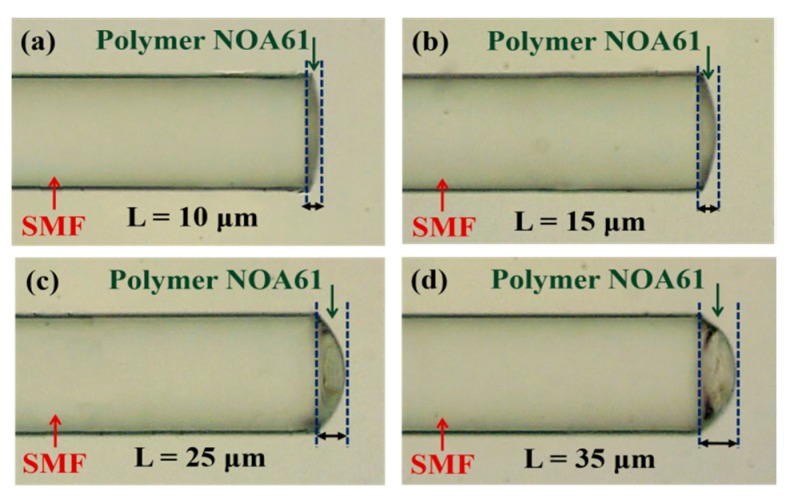
Micrographs of the proposed HPFFPIs with various hot-polymer lengths: (**a**) *L* = 10; (**b**) *L* = 15; (**c**) *L* = 25; (**d**) *L* = 35 μm.

**Figure 4:**
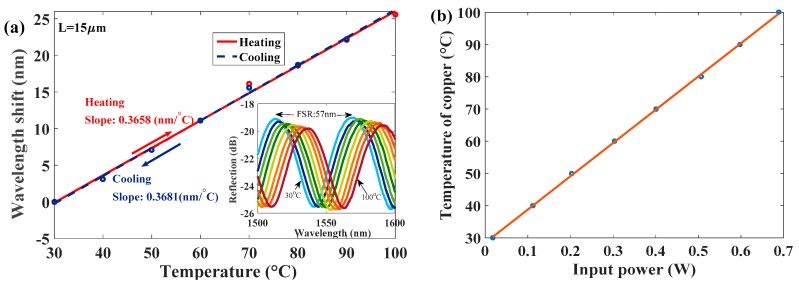
(**a**) Sensitivities of the proposed sensor to T during heating and cooling; inset is the corresponding spectrum; (**b**) T of the proposed heater corresponding to input power.

Figure 3 displays micrographs of the sensors with different cavity lengths (L) of (a) 10 μm, (b) 15 μm, (c) 25 μm, and (d) 35 μm. As the proposed anemometer relies on the measurement of the wavelength shift in response to changes of T that are caused by the airflow, the flow speed can be determined. First, to evaluate the superior characteristics of the response of the test HPFFPI where *L* = 15 μm to T, the aforementioned heater, made with a tiny and low-cost chip resistor + chip thermistor, is used to vary the temperature of the polymer head. The Fabry–Perot interferometer (FPI) interference fringes are expected to shift with the variation in T, and the fringes of the sensor have a periodical behavior. In the measurement, if the wavelength shift exceeds the Free Spectral Range (FSR) of the FPI fringes, the measured data cannot be identified. Therefore, the measurement range should be limited. However, in this study, the proposed polymer cavity is very tiny so as to produce a large FSR, as shown in the inset of Figure 4a. This is the advantage of the wide-range measurement. Figure 4a presents measurements of T, which are highly repeatable and responses are linearly proportional to T in the range of 30~100 °C. Figure 4b shows the T of the heater, estimated by the chip thermistor corresponding to the input power of the chip resistor. It can be seen in Figure 4a, regardless of whether the HPFFPI is being heated or cooled, that its thermal sensitivity is almost the same, and its highly linear performance demonstrates the excellent characteristics of the NOA61 polymer for the purpose of sensing parameters that are related to T. The high sensitivity arises from the high thermal expansion coefficient (TEC) of 2.2 × 10^−4^ °C^−1^ of NOA61 [15], which is greater than that of the silica fiber (∼5.5 × 10^−7^ °C^−1^). After the T sensing performance was evaluated, the proposed HPFFPIs with different values of L and input heating power (P) levels were used to measure the airflow.

**Figure 5:**
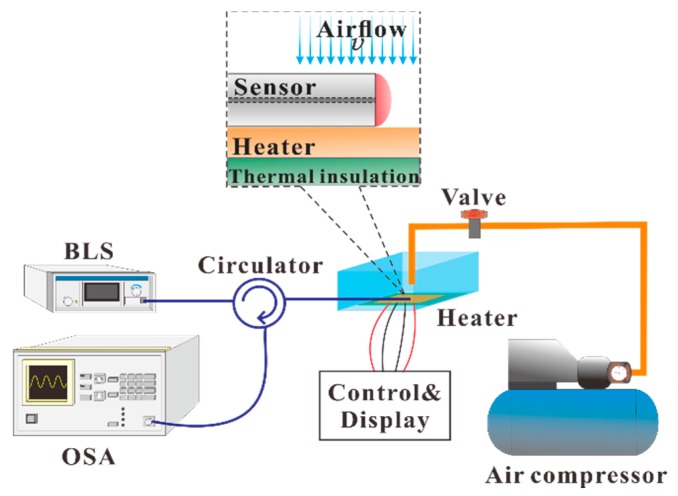
Experimental setup for measuring speed of airflow.

Figure 5 shows the experimental setup for making flow measurements. In this experimental case, when the chip thermistor achieved its steady-state T of 70 °C, the corresponding input power was measured to be about 0.4 W. Then, the sensor was close-packaged on thermal insulation plates, and only the top of the polymer head was exposed to air. The measurement system consists of an optical spectrum analyzer (OSA) (ADVANTEST-Q8384), a broadband light source (BLS) (Opto-Link Corporation Limited OLSWB-OESCLU-FA), and an optical circulator. The light propagates to the fabricated HPFFPI, which produces the quasi-sinusoidal interference patterns over a very wide range of wavelengths, which can be detected by the OSA. The HPFFPI was provided with airflow an air compressor, controlled by a tunable steady flow valve. For reference, the actual speed of the air was determined using a commercial hot-wire anemometer and found to be very close to that measured by the HPFFPI probe at the same level. The reflection spectra were individually recorded by the OSA when the speed of the airflow changed, affecting the hot polymer.

## 3. Experimental Results and Discussion

**Figure 6:**
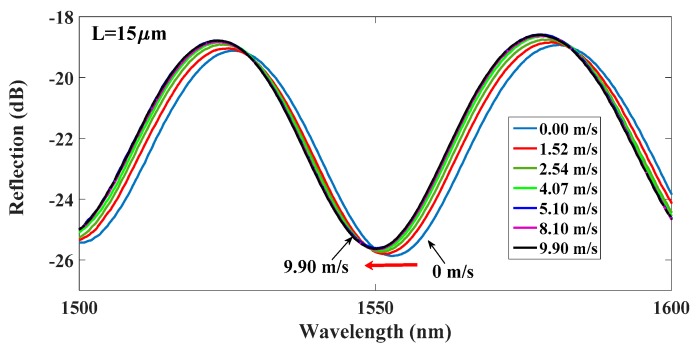
Reflection spectra of HPFFPI with *L* = 15 μm for various speed of airflow.

Exploiting the favorable repeatable T-responsive characteristics of the NOA61 polymer, the proposed HPFFPI was used to measure the airflow around the device to evaluate the effectiveness of the sensing scheme. Initially, the T of HPFFPI was effectively increased using the chip heater before the airflow was measured. The proposed tiny chip resistor was very cheap, keeping the cost of the sensing system very low. The proposed devices were placed on the heater with a high T, and surrounding’s relative humidity (RH) in our laboratory was around 50%. The air flowed immediately over the surface of heated polymer, reducing its T, blue-shifting the wavelength fringe valley of the interference spectra. Two beams of light from the interfaces of the polymer cavity yielded pure sinusoidal interference patterns over a wide range of wavelengths. The airflow sensing results in Figure 6 reveal that cooling reduces the T of the hot polymer and shrinks the polymer cavity, blue-shifting the wavelength fringe. In the experimental case, the input power to the chip resistor and polymer length L are 0.402 W and 15 μm, respectively.

The overall wavelength shifts at airflow speeds of 4.07 m/s and 9.90 m/s are 2.36 nm and 2.75 nm, respectively, for a fringe valley of around 1550 nm.

**Figure 7:**
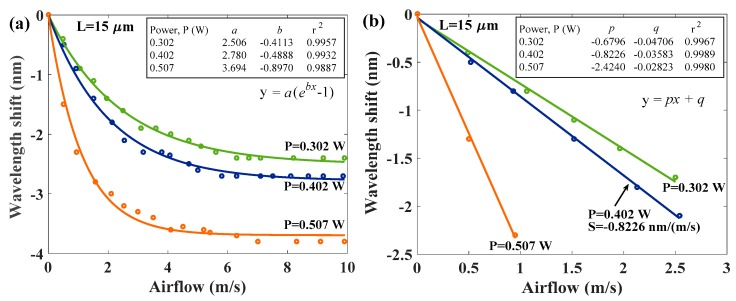
(**a**) Wavelength shifts of interference spectra of 15 μm HPFFPI under various airflows at different input power levels to the chip resistor; (**b**) Wavelength shifts of interference spectra of 15 μm HPFFPI under different input power levels of the heater.

Figure 7 shows the wavelength shifts of measured interference of HPFFPI anemometer with *L* = 15 μm in different input power to the chip resistor. An average measurement range of about 0~10 m/s is obtained. Wavelength shifts vary substantially to the airflow when the speed is below 5 m/s. However, the sensing response becomes weaker at higher airflow rates of v = 5~10 m/s, so measurements were not made beyond approximately 10 m/s. Based on the experimental measured data, we achieved fitting curves of wavelength shifts (Δλ) corresponding to speed of airflow (v) with an R-squared value (r^2^) above 0.99, as shown below:

(1) $$\Delta \lambda =a\left(e^{bv}-1\right)$$

here, *a* and *b* are the constants of exponential fitting curve. *b* especially is a negative value since the Δλ responses to the v presenting an exponential decay performance. From Equation (1), the speed of airflow (v) can be easily determined by using the following equation:

(2) $$v=\frac{1}{b}\ln\left(\frac{\Delta \lambda }{a}+1\right).$$

From Equation (2), one can estimate the speed of the airflow (v) when the wavelength shifts Δλ of the spectra are measured. The exponential fitting constants *a* and *b* for corresponding cases are shown in the inset of Figure 7a. In Figure 7a, the results demonstrate that a higher input power provides higher sensitivity. The highest exponential decay rate with the highest sensitivity of the wavelength shift $\Delta \lambda =3.698\left(e^{-0.897v}\mu 1\right)$ is obtained in the case of the input power of 0.507 W. We can see a linear response in the low airflow. The estimated linear sensitivities (S) with the linear fitting of y=px+q in the proposed 15 μm HPFFPI with different input power levels are plotted in Figure 7b. Here, p and q are the linear fitting constants. The linear S of 0.6796, 0.8226, and 2.424 nm/(m/s) are determined for the input power levels of *P* = 0.302 W, 0.402 W, and 0.507 W, respectively. The highest sensitivity of 2.424 nm/(m/s) is achieved at the *P* = 0.507 W condition, but the sensing range is very small (below 1 m/s) in the case due to its enormous exponential decay rate.

**Figure 8:**
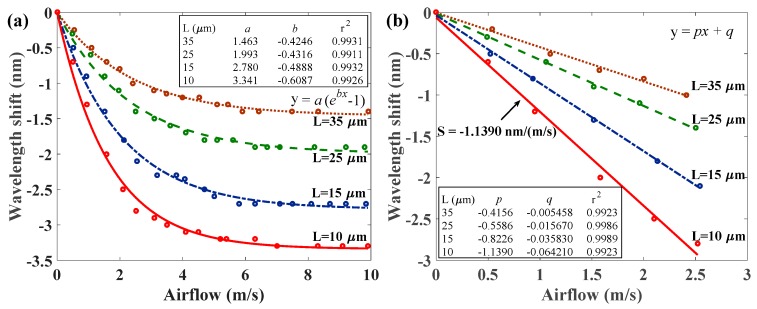
(**a**) Wavelength shifts of interference spectra of different HPFFPIs under airflows of various speeds when the hot polymer is initially heated with an input power of 0.402 W; (**b**) Measured sensitivities with linear responses at low airflow speed for different HPFFPIs (below 2.54 m/s).

Figure 8 presents sensing responses with lengths of the polymer cavity of *L* = 10, 15, 25, and 35 μm when selecting the input power of *P* = 0.402 W. It shows the wavelength shifts of the measured interference of HPFFPI anemometers with various L values as functions of the speed of airflow. The wavelength shifts also vary significantly with airflow (v), at low airflow speeds, and become weaker at higher airflow rates. The fitting curves of wavelength shifts (Δλ) corresponding to the speed of airflow (v) can also be expressed as Equation (1): $\Delta \lambda =a(e^{bv}-1$), those fitting constants *a* and *b* are shown in the inset of Figure 8a. The experimental results demonstrate that a shorter cavity of the polymer provides a higher sensitivity, and a poorer result is obtained as L increases. A much thinner cavity of the hot polymer is expected to provide greater sensitivity owing to its strong thermal transfer response. The highest exponential decay rate with the highest sensitivity of wavelength shift $\Delta \lambda =3.341\left(e^{-0.6087v}-1\right)$ is obtained in the case of *L* = 10 μm shown in the red line. The results of Figure 7a and Figure 8a demonstrate that the sensitivity of the proposed anemometer is quite high when the airflow is low and becomes more stable at high airflow levels. The dependence can be explained according to Newton’s law of cooling, which is demonstrated in [5]. Newton’s law of cooling states that the rate of change of the temperature of an object is proportional to the difference between its own temperature and the ambient temperature. On the other hand, it is also obvious that the chip thermistor with a fixed power level continuously heats the polymer to the extent that the temperature of the polymer cannot be substantially reduced, even at higher speeds of airflow.

Figure 8b plots the linear sensitivities (S) of the proposed HPFFPIs with different cavity lengths (L) to the low speed of airflow of the sensing range. The highest sensitivity of −1.139 nm/(m/s) = −1139 pm/(m/s) under the measured range of 0~2.54 m/s is also achieved at *L* = 10 μm. The measured reflection spectrum with a spectral resolution of the OSA of 0.01 nm yields a high resolution of 0.0088 m/s in the case of a 10 μm HPFFPI. Notably, the sensitivities of the proposed HPFFPIs that are caused by the high thermal expansion effect of the hot polymer would be larger than those of the reported hot FBG-based fiber-optic anemometers.

## 4. Conclusions

This work proposed the first tiny, simple, and low-cost fiber-optic anemometer that is based on a hot-polymer fiber Fabry–Perot interferometer (HPFFPI). This anemometer with the NOA61 polymer attached to its SMF endface was initially heated by a tiny chip resistor with a T that exceeds the temperature of the surroundings. The hot polymer was cooled by flowing air, greatly shifting the wavelength fringes of its interference spectra owing to its high thermal sensitivity. A high sensitivity of −1.139 nm/(m/s) and a high resolution of 0.0088 m/s were achieved when the length of the hot polymer was *L* = 10 μm and the heating input power was about 0.4 W. The heating power can be easily decreased when the size of the copper is reduced. Experimental results indicate that the proposed simple HPFFPI anemometer responds sensitively to variations in the rate of airflow, indicating that it can potentially be used as a fiber-optic anemometer.
